# Stage-Specific Expression Profiling of *Drosophila* Spermatogenesis Suggests that Meiotic Sex Chromosome Inactivation Drives Genomic Relocation of Testis-Expressed Genes

**DOI:** 10.1371/journal.pgen.1000731

**Published:** 2009-11-20

**Authors:** Maria D. Vibranovski, Hedibert F. Lopes, Timothy L. Karr, Manyuan Long

**Affiliations:** 1 Department of Ecology and Evolution, The University of Chicago, Chicago, Illinois, United States of America; 2 The University of Chicago Booth School of Business, Chicago, Illinois, United States of America; 3 The Biodesign Institute, Arizona State University, Tempe, Arizona, United States of America; Fred Hutchinson Cancer Research Center, United States of America

## Abstract

In *Drosophila*, genes expressed in males tend to accumulate on autosomes and are underrepresented on the X chromosome. In particular, genes expressed in testis have been observed to frequently relocate from the X chromosome to the autosomes. The inactivation of X-linked genes during male meiosis (i.e., meiotic sex chromosome inactivation—MSCI) was first proposed to explain male sterility caused by X-autosomal translocation in *Drosophila*, and more recently it was suggested that MSCI might provide the conditions under which selection would favor the accumulation of testis-expressed genes on autosomes. In order to investigate the impact of MSCI on *Drosophila* testis-expressed genes, we performed a global gene expression analysis of the three major phases of *D. melanogaster* spermatogenesis: mitosis, meiosis, and post-meiosis. First, we found evidence supporting the existence of MSCI by comparing the expression levels of X- and autosome-linked genes, finding the former to be significantly reduced in meiosis. Second, we observed that the paucity of X-linked testis-expressed genes was restricted to those genes highly expressed in meiosis. Third, we found that autosomal genes relocated through retroposition from the X chromosome were more often highly expressed in meiosis in contrast to their X-linked parents. These results suggest MSCI as a general mechanism affecting the evolution of some testis-expressed genes.

## Author Summary

During the course of *Drosophila* evolution, genes expressed in males have accumulated on the autosomes. Meiotic sex chromosome X inactivation in males was proposed, among other hypotheses, as a selective force favoring the accumulation of testis-expressed genes on the autosomes. Under such a model, the inactivation of X-linked genes would favor the accumulation of testis-expressed genes in autosomes, wherein these genes would still be expressed. In this study, we observed meiotic expression reduction for X-linked genes in *D*. *melanogaster* through a global gene expression analysis in different phases of spermatogenesis, in agreement with MSCI. In order to test the effects of MSCI on the chromosomal distribution of testis-expressed genes, we analyzed their expression pattern throughout spermatogenesis. First, X chromosome underrepresentation was restricted to testis-biased genes over-expressed in meiosis. Second, we observed that the autosomal genes retroposed from the X chromosome more often showed complementary expression in meiosis to their X-linked parents. These results support MSCI in *Drosophila*, suggesting its mechanistic role in the evolution of testis-expressed genes.

## Introduction

Sex chromosomes evolve differently than autosomes due to their distinct characteristics such as a lack of recombination between the homologues (X and Y), a different number of chromosome copies between sexes, and the proportion of heterochromatin [1]. Genomic and proteomic studies in *Drosophila*, mammals and worms reveal that male-biased genes, i.e. those that are more highly expressed in males than in females, are underrepresented in the X chromosome [2]–[7]. One possible mechanism contributing to this biased chromosomal distribution is the directional movement of testis-expressed genes out of the X chromosome. In *Drosophila* and mammals, there is a significant excess of genes retroposed from the X chromosome to the autosomes, and these genes are more likely to be expressed in testis [8]–[11]. Recently, in *Drosophila*, this observation was found to also apply for DNA-based gene duplication [12],[13].

Two evolutionary hypotheses have been proposed to explain the underrepresentation of genes expressed in males on the X chromosome. First, the meiotic sex chromosome inactivation (MSCI) hypothesis, can explain both the excess of retroposed genes from the X chromosome to the autosomes as well as the paucity of X-linked testis-expressed genes [9],[14]. According to this hypothesis, the inactivation of X-linked genes during male meiosis favors the accumulation of testis-expressed genes in autosomes where such genes can be expressed during the meiotic stage [14].

A second hypothesis, based on a model proposed by Rice [15] and by Charlesworth and co-authors [16], states that sex biased expression is driven by sexually antagonistic forces, i.e., the case of opposing selection pressure on the two sexes. In this model developed through the investigation of polymorphic equilibrium, dominant mutations with beneficial fitness effects in females, but detrimental effects in males, will have a higher probability of being fixed on the X chromosome [15], while recessive sexually antagonistic mutations will have a higher probability of being fixed on the autosomes [16]. The opposite pattern is expected for male-beneficial, female-detrimental mutations: dominant and recessive alleles will have higher probability of being fixed on the autosomes and on the X chromosome, respectively [15]. Recently, however, Patten and Haig [17] showed that male-beneficial mutations could also be fixed on the X chromosome even for some cases of dominant alleles. In addition, is important to note that Gibson and colleagues' theoretical and empirical findings [18] also suggest the X chromosome to be enriched in polymorphism for sexually antagonistic alleles.

In order to relate the sexual antagonism hypothesis to the male-biased gene chromosomal distribution, one must assume a model under which sexually antagonistic fitness variation contributes to sex-biased expression. In the model proposed by Rice [15], the paucity of X-linked male-biased genes would be a consequence of restrictive conditions for sexually antagonistic mutations. For instance, the model requires that: i) the majority of X-linked sexually antagonistic mutations are female-beneficial/male-detrimental; or ii) most male-beneficial/female-detrimental alleles are dominant [for a review, see [19]]. Although none of these requirements have been tested yet, the sexual antagonism hypothesis could explain the underrepresentation of male-biased genes on the *Drosophila* X chromosome [3],[20]. Most important, the observation of similar X chromosome underrepresentation found for somatic cells revealed that the chromosomal distribution of male-biased genes cannot be exclusively explained by MSCI [3],[20].

MSCI appears to be phylogenetically plastic: it has been observed in the X chromosome of male mammals and nematodes [21],[22], and in the Z chromosome of female birds [23]. Meiotic X chromosome inactivation in *Drosophila* males was first suggested as the cause of male sterility in mutants carrying X-autosomal translocations [14]. Recently, MSCI was investigated by inserting transgenic constructs expressing a testis-specific promoter, into different regions of the genome [24]. A reduction in the expression of reporter gene insertions into the X chromosome was observed, consistent with a MSCI model for *Drosophila*. In contrast to this result, however, a microarray-based study of male germline expression using spermatogenic arrest mutants did not detect a significant signal of MSCI [20].

In order to investigate the impact of MSCI on *Drosophila* testis-expressed genes, we conducted a global gene expression analysis of spermatogenesis in a developmental context. We compared X and autosome expression in three distinct regions of the testis, (the apical, mid- and posterior testis), that are correspondingly enriched with mitotic, meiotic and post-meiotic cells. It is important to note that in mammals, different studies [6],[25] have shown variability in gene expression reduction as a result of MSCI (e.g., a 60% to 80% X-linked expression reduction was observed in microarray-based experiments, [25]). In order to avoid an absolute definition of completely silenced expression, here we defined MSCI as a significant expression reduction in the male germline X chromosome.

Our results clearly show a small, but significant, reduction of X-linked gene expression in meiosis, consistent with MSCI. We also investigated the impact of MSCI on the chromosomal distribution of testis-expressed genes by analyzing their expression profile during spermatogenesis. MSCI was positively correlated with both the retrogene chromosomal movement and the underrepresentation of X-linked testis-biased genes. Our findings suggest MSCI as a general mechanism affecting the evolution of some testis-expressed genes.

## Results/Discussion

### Global gene expression analysis during spermatogenesis

**Figure 1:**
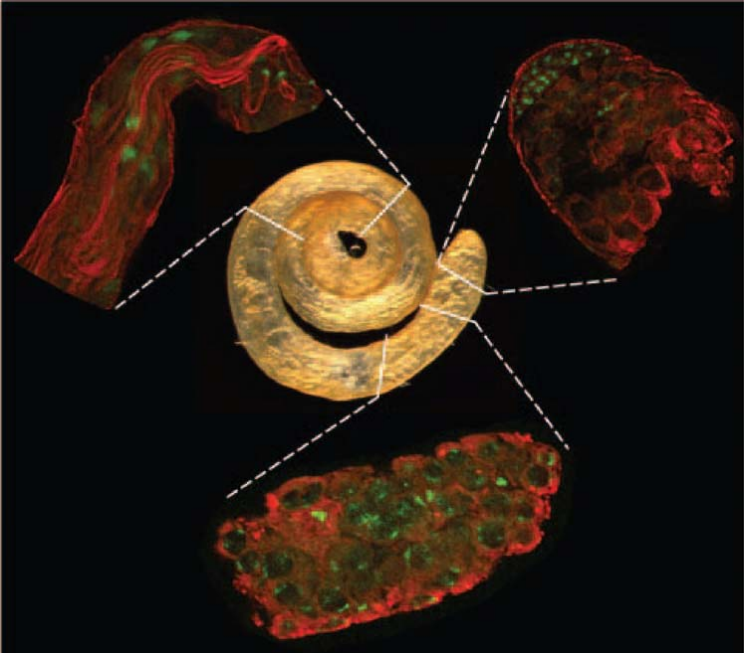
Spermatogenic tissues. Whole testis (center image) and isolated tissue samples (peripheral images in clockwise direction starting in the upper right): apical tip, proximal, and distal regions enriched with mitotic, meiotic and post-meiotic phases, respectively. Indirect immunofluorescence staining is shown in red for α-tubulin and in green for DNA. Shape and position of those cell elements differentiate cell types such as mitotic cells, spermatocytes and elongated spermatozoids.

To measure MSCI and investigate the impact of X inactivation on testis-expressed genes, we conducted a genome-wide expression analysis of wild-type males using three cell populations isolated from mitotic, meiotic and post-meiotic phases of spermatogenesis in *Drosophila melanogaster*, respectively (Materials and Methods, Table S1). Figure 1 details the precise regions of the testis that were isolated in this study. According to MSCI in mammals, we expected to find in *Drosophila* differential X-linked expression specific to spermatogenic meiotic cells [14],[20],[21],[24]. Therefore, our method allowed us to effectively measure the relative expression levels among the three phases and confirm MSCI.

Purification of *Drosophila* spermatogenic cells is not as straightforward as it is in mammals, where purified samples of specific germline phases can be isolated [6]. Therefore, our approach was to directly isolate testis regions enriched with RNAs from each of the three specific germline phases (Figure 1, Materials and Methods). While the cell types present at various stages of spermatogenesis are generally located in a gradient along the proximal-distal axis of the testis, most are not exclusively restricted to any one geographic region. Therefore, we used the cellular morphology of the three phases (Figure 1) as a guide to isolate cells enriched for the following cell populations: i) *apical* (*mitotic*) – enriched for apical hub stem cells, spermatogonial cysts with reduced levels of primary spermatocyte cysts; ii) *mid-region* (*meiotic*) – enriched for primary and secondary spermatocyte cysts; reduced levels of early spermatids; iii) *distal* (*post-meiotic*) – enriched for elongating spermatid cysts, individualized spermatid bundles and coiled spermatozoa, reduced levels of spermatocyte cyst cells. Careful dissection of these landmark regions produced exceptional reproducibility of the data as confirmed by the high correlation within replicates from the same spermatogenic phases (r 0.993, Figure S2).

**Figure 2:**
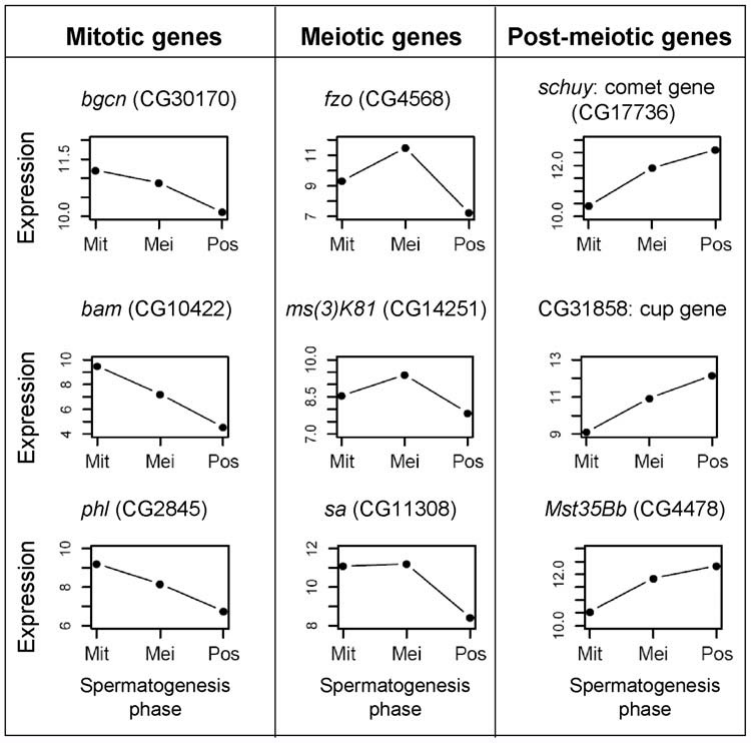
Expression profile for spermatogenic genes. Examples of expression observed in our database for genes known to be involved in mitotic, meiotic and post-meiotic spermatogenic processes. X axes: Mit stands for mitosis, Mei for meiosis and Pos for post-meiosis. Expression is given as microarray intensity (log2). Gene symbol: *bgcn*, *benign gonial cell neoplasm*; *bam*, *bag of marbles*; *fzo*, *fuzzy onions*; *phl*, *pole hole*; *sa*, *spermatocyte arrest*.

In order to evaluate the cell content of mitotic, meiotic and post-meiotic samples, we examined our dataset for individual genes known to be expressed in specific spermatogenic stages. For instance, genes known to be involved in early spermatogenic processes are highly expressed in the mitotic phase, followed by decreased expression in meiosis and post-meiosis (Figure 2). For example, *bag-of-marbles* and *benign gonial cell neoplasm* genes act early in the *Drosophila* germ cells and their mutations prevent progression through spermatogenesis and cell differentiation [26]. Both genes were over-expressed in our mitotic sample (Figure 2), which, in overall, showed enrichment for genes involved in early spermatogenic processes (Fisher exact test, P 0.014). On the other hand, genes expressed in spermatocytes, such as *K81*, showed a peak of expression in meiosis (Figure 2). *K81* is a paternal effect gene showing a post-fertilization phenotype but known to be exclusively expressed in primary spermatocytes [27]. Additionally, genes such as *schuy* and CG31858 showed low levels of mitotic expression followed by an increase in expression during meiosis, but peaking in the post-meiotic phase (Figure 2). Using *in situ* hybridization, a recent study showed that these genes are transcribed in post-meiosis [28]. These genes have been categorized and named as *comet* and *cup* genes according to their transcript localization patterns, since both are found in the end of elongated spermatids [28].

In summary, our samples were enriched with stage-specific cells and gene expression profiles can be accessed via the public database SpermPress (http://pondside.uchicago.edu/~longlab/spermpress). While our samples do not perfectly separate the three phases of spermatogenesis, our cell enrichment technique and the high degree of reproducibility in the replicate datasets should be enough to detect the reduction of X-linked expression during meiosis, as expected under MSCI.

### MSCI in *D. melanogaster*

The existence of MSCI in *Drosophila* was recently investigated using reporter gene constructs whose expression was driven by a testis specific promoter [24]. In this study, the authors observed a significant reduction in the expression of constructs inserted into the X chromosome when compared to those inserted into the autosomes, and proposed this was consistent with the existence of MSCI. In contrast, a recent microarray survey of male germline expression using spermatogenic arrest mutants found no evidence for MSCI [20]. However, the mutants used in this study were completely depleted of later meiotic cells, compromising the overall investigation of meiotic MSCI [20].

We decided to directly confirm meiotic effects of MSCI in *D. melanogaster* by comparing the expression profile of the X chromosome and autosomes during the three stages of spermatogenesis. MSCI leads to a clear expression prediction: there should be a significant decrease in the expression of X-linked genes when compared to autosomal-linked genes exclusively during the meiotic phase.

**Figure 3:**
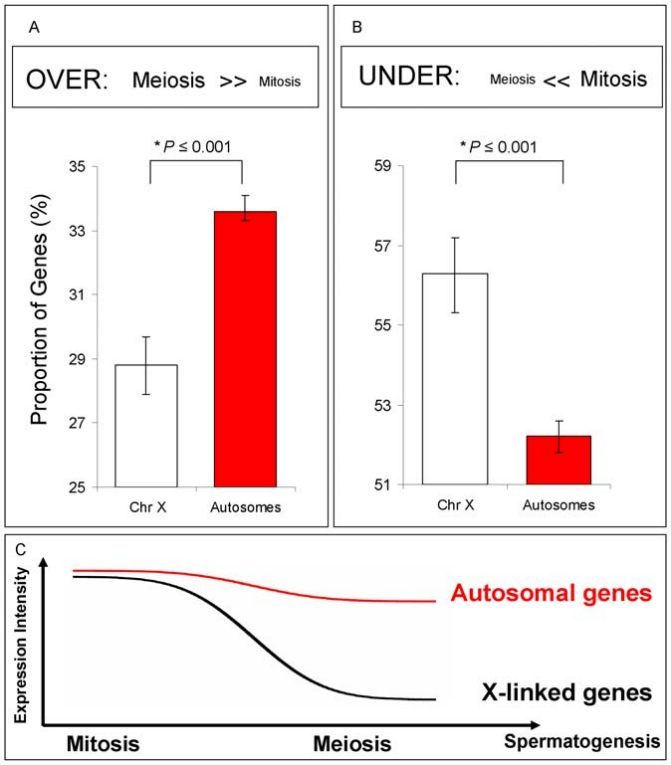
Spermatogenic gene expression for X-linked and autosome-linked genes. Mitotic and Meiotic relative profiles for genes known to be expressed in testis (Flyatlas) (A) and (B) Proportions of genes and their respective Bayesian 95% Confidence Intervals in each of the following classes: (A) Genes over-expressed in meiosis (expression in meiosis >> expression in mitosis); (B) Genes under-expressed in meiosis (expression in meiosis << expression in mitosis). For all comparisons (A) and (B), X chromosome gene proportions are significantly different than autosomal gene proportions (*P stands for *Bayesian P*, Methods in Text S1). (C) Spermatogenic diagram summarizing expression results in (A) and (B). A significant proportion of X-linked genes has reduced expression in meiosis, but not in mitosis.

Conventional microarray analyses based on average expression intensities, such as the one previously used to investigate the existence of X inactivation [20],[29] lose important information (such as variation and distribution of the expression signals), potentially compromising statistical hypothesis testing. Such limitations may be particularly important regarding the X chromosome expression reduction expected during MSCI [20]. There are two main statistical concerns we aimed to address: 1). how to deal with the variation unavoidably introduced by factors such as expression fluctuation among repeated experiments; and 2). how to deal with the small proportion of non-germline cells mixed in our samples. To approach these two issues, we introduced Bayesian statistical models for analyzing our data and testing the hypothesis of MSCI. This approach permitted the use of the data in its entirety to simultaneously estimate proportions of under-, over- and equally expressed genes in meiosis, while comparing X chromosome and autosomal distributions (Model A in Statistical Analyses, Methods in Text S1; Figure 3) [30]. The test avoided the specification of arbitrary thresholds commonly employed such as fold change methods [3],[31]. It also eliminated errors introduced by multiple hypothesis testing [32].

Our Bayesian approach modeled the proportions of genes from the X chromosome and autosomes that were over-, under- and equally expressed in meiosis relative to other spermatogenic phases. X inactivation could thus be observed as an excessive number of X-linked genes under-expressed in meiosis, *i.e.*, with expression reduction in meiosis. On the other hand, if the X chromosome is active, we would expect to observe no difference between autosomal and X chromosome proportions of over-, under- and equally expressed genes.

We restricted our analyses to those genes that had been previously shown to be expressed in whole testis microarray experiments (Flyatlas presence call, Table S1). In our genome-wide phase analysis, we detected a significant under-expression of X-linked genes compared to autosome-linked genes in meiosis relative to mitosis (Figure 3A and 3B). In the meiotic phase, the proportion of over-expressed X-linked genes was significantly reduced when compared to the autosome-linked genes (Figure 3A; Bayesian P≤0.001), whereas the proportion of under-expressed X-linked genes was increased, as indicated by the distribution of X-linked and autosome-linked genes (Bayesian P≤0.001) (Figure 3B). The difference between the proportion of autosome-linked and X –linked genes under-expressed in meiosis (Figure 3B) is in agreement with the expression reduction of the X chromosome in meiosis expected by our previous definition of MSCI (Figure 3C). Similar results were obtained when using the complete dataset of genes, instead of only those already known to be expressed in testis (Figure S3C).

The clear separation of chromosome proportions for each class detected by our Bayesian model contrasts with the results obtained using an arbitrary twofold change threshold in the same data (Figure S3D, Methods in Text S1). This is a result of the increased power of the Bayesian approach for detecting genome-wide expression differences and of the negligible within-gene variability compared to between gene-variability produced by our experimental replicates.

Although we observed a significant reduction in meiotic expression on the X chromosome, it is clear that such reduction does not meet the textbook definition of “X inactivation”, that is, the complete silencing of expression. Indeed, we observed a fairly small (10%) average X-linked gene meiotic expression reduction when compared to genes in the autosomes, which does not indicate whole-scale inactivation of the X (94% vs. 84% for autosomal- and X-linked genes, respectively).

Moreover, the reduction observed in our experiments is considerably different (shows lower reduction magnitude) from the one obtained by Hense and co-authors using transgenic construct insertions [24]. The expression of transgenes inserted into the X observed by Hense *et al*., however, was universally reduced (between 3.4 to 10-fold) compared to transgenes inserted into the autosomes [24]. There are differences in the experimental properties that could account for the discrepancies obtained by the two experiments. First, Hense and co-authors analyzed construct insertions for the promoter of a gene highly and specifically expressed in testis [24], whereas our analysis includes the expression profile of all *D. melanogaster* gene products. This difference *per se* could generate discrepant observations of expression reduction. Indeed, Hense and co-authors [24] commented that their system may be especially sensitive for detecting X-inactivation because they used a minimal promoter from a gene that appears to be expressed relatively late during spermatogenesis. Second, the two studies used different methods to measure RNA intensity. Hense *et al*. measured RNA level by -Galactosidase activity and qRT-PCR, whereas we used a microarray technique [24]. These methods can present differences in resolution and expression variation. For instance, Hense and co-authors observed a lot of variation in expression reduction of the X chromosome, depending on the method used: -Galactosidase activity showed a 10-fold difference, whereas qRT-PCR showed 3.4-fold difference [24]. In our experiments, genes found to be under-expressed in meiosis showed an average two-fold expression reduction in meiosis compared to mitosis (Figure S4). However, individual gene expression reduction varied from 1.068 to 17.70 fold. Third, the heterogeneous cell makeup in our experiments could have obscured the signal on the microarray. Our samples were enriched with cells from different stages and therefore do not represent pure cell types. Other possible discrepancies also could account for our different results (e.g. transgene insertions can be affected more strongly by different chromatin structures, or X chromosome expression reduction can arise during later phases of meiosis).

Our global gene expression analysis, however, clearly showed that not all X-linked genes were affected in the same way, i.e., not all X-linked genes were under-expressed in meiosis. The fact that our samples were enriched with cells from different spermatogenesis stages rather than corresponding to pure samples could explain this result. On the other hand, MSCI in *Drosophila* might not be globally homogeneous, and therefore might be similar to what occurs in mammals [33].

Despite the discrepancies between our results and those of Hense *et al*. [24], it is important to note the similarities between them. Both showed a significant X chromosome expression reduction in male meiosis. The two studies used different yet complementary approaches and methods but reached similar conclusions. Our results form an independent and direct genomic test for the hypothesis of MSCI, which was indirectly supported by Hense and co-authors [24]. Moreover, the large-scale gene expression data of male meiosis provided by our study thus allowed us to investigate the evolutionary effects of meiotic expression reduction on X-linked testis-expressed genes, such as retrogene chromosomal relocations (see below).

The X chromosome has a paucity of testis male-biased genes (most likely male-biased germline cells) [3]–[5], and could be consequently enriched with non male-biased testis somatic genes. Therefore, one could argue that a lower proportion of somatic tissue in our meiotic sample (for example, absence of testis sheath), when compared to the mitotic sample, could account for the lower expression of X-linked genes. There are at least two arguments against this interpretation. First, most of the transcription in the testis occurs in germline cells and therefore somatic expression is unlikely to make a significant contribution [34]. Second, the paucity of male-biased X-linked genes was also observed for somatic tissues (in gonadectomized males) and therefore somatic testis genes probably show the same expression pattern [3]. Therefore, in all likelihood, differences in the amount of somatic and germline cells in our samples, did not make a major contribution to chromosomal expression differences.

**Figure 4:**
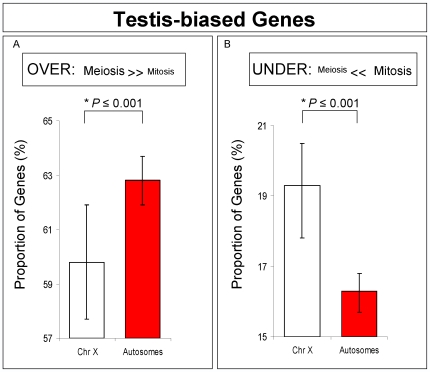
Testis-biased genes expression for X-linked and autosomal-linked genes. Proportions and Bayesian 95% Confidence Intervals of testis-biased genes over-expressed (A) and under-expressed (B) in meiosis. A significant proportion of X-linked genes has reduced expression in meiosis, but not in mitosis. *Bayesian P.

Nonetheless, we decided to further investigate if the difference we observed in chromosomal expression was due to a somatic bias in our samples. We compared X chromosomal and autosomal expression exclusively for testis-biased genes selected from previous gonad comparison experiments [3],[4]. Using this approach, we were able to exclude the contribution of somatic genes. In complete agreement with our previous observations, we detected an excess of X-linked genes under-expressed in meiosis, thus providing supporting evidence for MSCI (Figure 4).

An alternative hypothesis to MSCI for our expression results is that the lowered expression of X chromosome genes in the meiotic phase is due to a loss of dosage compensation, which in *Drosophila* is achieved by hypertranscription of the X chromosome in males [35]. Previous analyses comparing ovary and testis expression have already established the existence of dosage compensation in the male germline [35]. Therefore, taking into account our results, it is unclear why dosage compensation should be limited to a period of the entire spermatogenesis process. The lower level of X chromosome expression restricted to the particular meiotic stage that we observed, however, is consistent with male germline X inactivation. Another piece of evidence against loss of dosage compensation related to lower expression in X-linked testis expressed genes was obtained by MSCI experiments done by Hense and co-authors [24]. In their work, the expression levels of transgenic constructs driven by a testis promoter inserted into the X were reduced compared to the same constructs inserted in only one of the homologous autosomal chromosomes (heterozygous insertion). If the phenomenon was due to loss of dosage compensation instead of MSCI, X-linked transgenic constructs should have been expressed at least at the same level as autosomal insertions, which was not observed [24].

At first glance, our global expression experiments and results are not able to empirically differentiate between MSCI and an *ad hoc* hypothesis of stage-specific loss of dosage compensation. If in fact there were a loss of dosage compensation occurring in meiosis, the results presented in the following sections would not change significantly. X chromosome down regulation in meiotic cells is associated with and impacts the general distribution of testis- biased genes and retrogenes.

Although we observed a significant reduction in expression for the X-linked loci in the meiotic sample when compared to the post-meiotic phase (Figure S5), we had hesitated to adopt an MSCI-based interpretation of this result. One reason for caution is that autoradiographic studies of *Drosophila* spermatogenesis have shown the absence of transcription in the post-meiotic phase [*e.g.*, [34]]. The prevailing notion has been that most proteins required in the late stages of spermatogenesis are translated from mRNAs produced during early meiosis and stored in the cytoplasm [34],[36]. However, a recent study using *in situ* hybridization provided compelling evidence of post-meiotic transcription for 24 selected *Drosophila* genes [28]. The ambiguity associated between post-meiotic translation and transcription could confound any observations regarding the prevalence of X chromosome inactivation after meiosis.

### MSCI and retrogenes movement out of the X chromosome in *Drosophila*

MSCI has been proposed to explain the accumulation of testis-expressed genes in autosomes [3],[9],[14]. Two specific predictions result from this hypothesis, one related to retrogenes, and the other related to testis-biased genes (see next section for the latter). The retrogene-based prediction refers to the disproportionate retroposition of X-linked genes to the autosomes, as a consequence of natural selection for expression of testis functions during MSCI [8],[9]. This prediction generates two testable expectations: (i) most autosomal retroposed genes that originated from X-linked parental genes should be over-expressed during the MSCI stage (i.e. meiosis); and (ii) retroposed genes should display complementary expression patterns during meiosis in relation to their X-linked parental genes. With our data, we have tested and confirmed both expectations.

**Figure 5:**
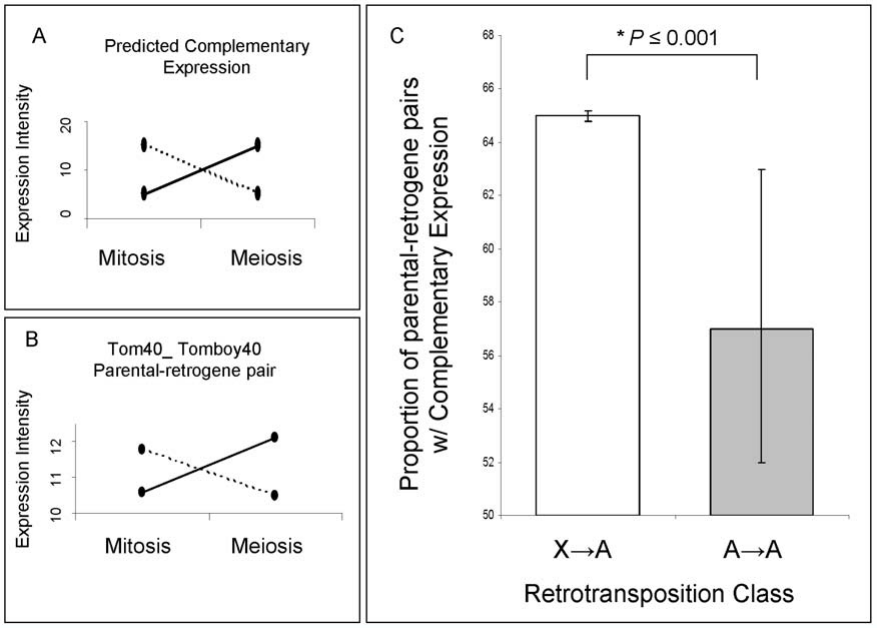
Parental gene and retrogene expression analysis. (A) Predicted complementary expression in meiosis is defined as under-expressed parental gene (dotted line) and over-expressed retrogene (solid line). (B) Empirical complementary expression pattern of the parental gene *Tom40* and the retrogene *Tomboy40* retroposed from X chromosome to autosome (Figure 6A and Table S2—pair 18) [10]. A and B vertical axes represent the gene expression intensities in log2, whereas the horizontal axes indicate spermatogenic phases. (C) Proportions and Bayesian 95% Confidence Intervals of parental-retrogene pairs satisfying complementary expression in meiosis: X→A pairs (white box), A→A pairs (grey box). *Bayesian P≤0.001.

**Figure 6:**
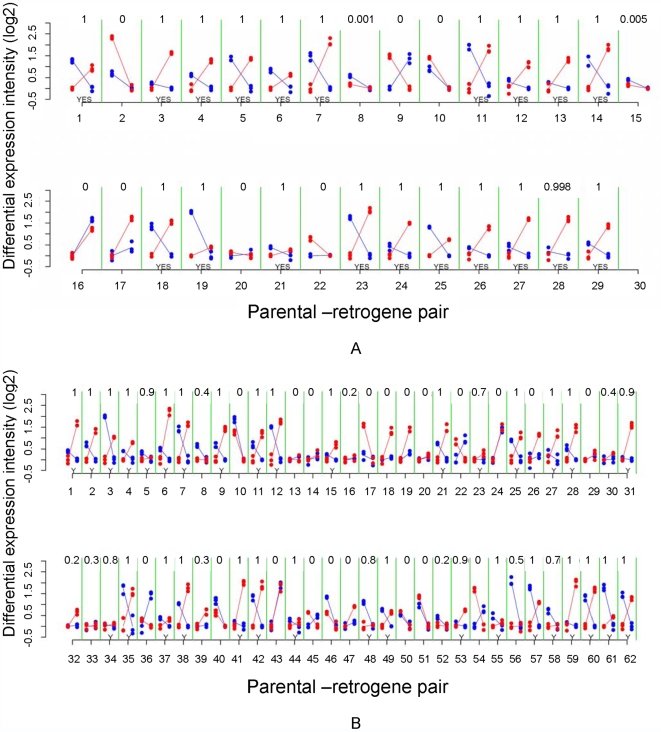
Mitotic and meiotic expression of individual parental-retrogene pairs. (A) Pairs retroposed from X chromosome; (B) Pairs retroposed from autosomes. Differential gene intensity stands for a difference in expression intensities between meiosis and mitosis. Parental genes and retrogenes intensities (log2) are shown in blue and red dots, respectively. Solid lines connect mitosis and meiosis intensity averages of three replicates. The X-axis numbers correspond to parental-retrogene pairs ordered according to Table S2. The numbers on the top of each parental-retrogene segment (between green lines) correspond to the posterior Bayesian probabilities that a pair shows complementary expression (Model B in Statistical Analyses, Methods in Text S1). The bottom yeses (Y) indicate genes where those probabilities are above 50% (Statistical Analyses, Methods in Text S1). Most of X→A parental-retrogene pairs (A) show either a probability of zero or one for complementary expression, whereas several A→A parental-retrogene pairs (B) have probabilities that are markedly different from zero or one (e.g., 0.7 and 0.4). These uncertainties when classifying genes directly affects the confidence intervals, which are much larger for A→A than for X→A parental-retrogene pairs (Figure 5C).

We observed that approximately 63% of all retroposed genes were over-expressed during meiosis in comparison to mitosis, where X inactivation occurs. As observed for mammals retrogenes [37], both autosomal copies retroposed from the X and copies retroposed from other autosomes are more often over-expressed in meiosis than non-retrogene autosomal genes (32%, n = 7827). This indicates that any autosomal retrogene is more likely to be expressed in meiosis. One possible explanation is that the “hypertranscription” state of autosomal chromatin in meiotic cells might facilitate the expression of young retrocopies soon after their emergence [37]. However, confirming the first expectation, autosomal copies retroposed from the X chromosome were more often over-expressed in meiosis than genes retroposed between autosomes (gene proportions: 71% versus 58%, Bayesian P≤0.002). To test the second expectation, we measured the complementary expression of parental-retrogene pairs in meiosis, i.e. parental gene under-expression and retrogene over-expression in meiosis (Figure 5A and 5B). Specifically, we compared the expression of 27 X→A and 52 A→A parental-retrogene pairs [10] in the mitotic and meiotic phases (Figure 6, Table S2). An extension of the previous Bayesian analysis (Model B in Statistical Analyses, Methods in Text S1) revealed that, compared to A→A retrogenes, X→A retrogenes had a significantly higher proportion of complementary expression in meiosis, thus confirming the second expectation (Figure 5C, Bayesian P≤0.001).

The complementary expression pattern strongly suggested that MSCI occurs specifically during the meiotic phase, and revealed a spatial and temporal link between over-expression of the retrogene and inactivation of the parental gene on the X chromosome during meiosis. In addition, the observation of higher meiotic expression of genes retroposed from the X chromosome showed that the complementary expression pattern does not solely result from the inability of parental X-linked genes to evolve higher levels of meiotic expression. The most parsimonious scenario is that autosomal retrogenes are favored because they recover the function of an X-linked parental downregulated copy.

As mentioned in the previous section, our global transcriptome analysis used samples enriched for three phases of spermatogenesis, but also included accompanying somatic cell types, which could bias the expression profiles obtained. Additional evidence against a bias created by non-germline cells comes from the specific effects of MSCI on retrogenes. Reduced somatic contribution in our meiotic sample (e.g., absence of testis sheath) could potentially account for the excess of over-expressed autosomal genes. However, our analysis showed that two different groups of autosomal genes had significantly different expression patterns in meiosis. Autosomal genes retroposed from the X chromosome showed a significantly higher meiotic expression than those retroposed from the autosomes (71% versus 58%, Bayesian P 0.002). Such a difference would not be expected if the somatic contribution in our samples was a major factor or the expression profile is an artifact in our analysis. MSCI impacting testis-expressed genes is a more plausible explanation.

### MSCI and the chromosomal distribution of *Drosophila* testis-biased genes

The existence of MSCI leads to another expectation: male-biased genes (those more highly expressed in males than in females) should accumulate in autosomes and, consequently, be underrepresented in the X chromosome [2]–[5]. There are no studies showing an effect of MSCI on the chromosomal distribution of male-biased genes. On the contrary, previous studies have shown that male-biased genes expressed not only in testis, but also in somatic cells are underrepresented in the X chromosome, which would suggest an explanation beyond MSCI (e.g., sexual antagonism or population genetic forces) [3],[20]. However, it is important to note that, in these studies, the proportion of somatic male-biased genes is an order of magnitude lower than the proportion of testis male-biased genes (<2% vs. <20%, Figure 2 in [3]), which would still allow an important role for MSCI.

**Figure 7:**
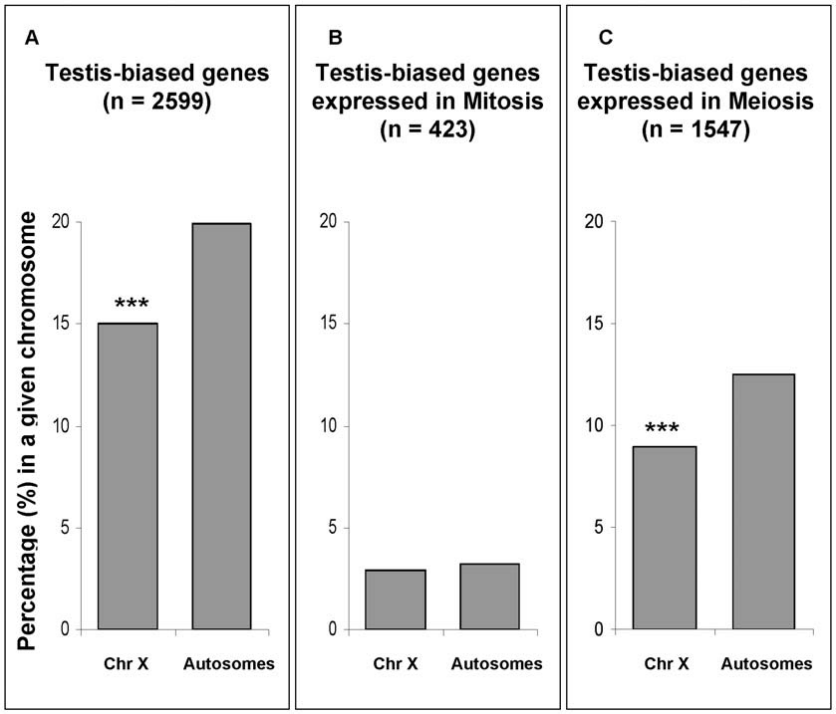
Chromosomal percentages of testis-biased genes. In gray, percentage of testis-biased genes in a given chromosomal location, i.e. autosomes and X chromosome (Chr X). (A) Percentages of all testis-biased genes regardless their spermatogenic phase of expression; (B) percentages of testis-biased genes over-expressed in mitosis; and (C) percentages of testis-biased genes over-expressed in meiosis (C). Sample sizes (n) for each group of testis-biased genes are shown in parentheses. For instance, panel (A) shows that approximately 15% of all X-linked genes are testis-biased (85% are non-testis-biased). Panel (B) and (C) are subsets of all testis-biased genes (panel A). Significant deviations (Fisher exact test) are indicated by ***P 10^−5^.

In our expression comparison between meiotic and mitotic phases, approximately 60% of testis-biased genes (those more highly expressed in testes than in ovaries) were over-expressed in the meiotic phase (Figure 7C, Table S3). In order to test for the effect of MSCI on the chromosomal distribution of testis-biased genes, we analyzed the effects of mitosis and meiosis on the chromosomal distribution of testis-biased genes (Figure 7). The underrepresentation on the X chromosome of testis-biased genes (Figure 7A) is only observed for genes over-expressed in meiosis (Figure 7C) (Fisher exact test, P≤0.001). Testis-biased genes highly expressed in mitosis were not depleted in the X chromosome (Figure 7B). Our results show that both X chromosome inactivation and X-linked testis-biased gene underrepresentation occur only in meiosis. Therefore, it seems that the paucity of testis-biased genes on the X chromosome is affected by MSCI.

Our testis-biased gene analysis reveals a different chromosomal distribution of mitotic genes than the one observed using *Drosophila* spermatogenic arrest mutants [20]. In that study, genes expressed in mutant testes, enriched with mitotic cells, were found to be underrepresented in the X chromosome [20]. An important difference in our approach as opposed to the mutant analysis [20] was our ability to directly detect and compare gene expression levels (both higher and lower) in mitosis versus meiosis, instead of measuring gene expression levels only in mitotic cells in the mutant testis. Our analysis shows that there is not a paucity of X-linked genes that are more highly expressed in mitosis, therefore indicating the effects of MSCI. Another feature of our direct approach of global expression analysis of wild-type testis was that it obviated problems associated with the use of mutants, such as concomitant pleiotropic and physiological effects that may affect gene expression patterns.

### Conclusion

In summary, our study coupled a global spermatogenic analysis with a Bayesian statistical method that overcame the limitations of conventional microarray comparison based on average expression intensities. We were able to detect significant expression reduction of X-linked genes during male meiotic phases. Although such reduction does not meet the absolute definition of gene silencing that may be unrealistic [25],[33], our results are in agreement with the detection of meiotic sex chromosome inactivation in males given the experimental methods currently available.

Our analysis also revealed a significant correlation between MSCI and retrogene chromosomal movement. Further, testis-biased genes are over-expressed during MSCI and the X-linked reduction is associated with the chromosomal distribution of those genes. These results suggest a critical impact of MSCI on the evolution of sex chromosomes in *Drosophila*. For example, the complementary nature of parental/retroposed gene pair expression during meiosis (Figure 5) illustrates the effects of MSCI on the origin, evolution and chromosomal localization of new testis-expressed retrogenes.

Previous studies using *Drosophila* ancestral X chromosome [9],[12],[13 and this work] and *D. pseudoobscura* neo-X chromosome [12],[13],[20] suggested that movement off of the X chromosome had significant role in reducing the proportion of male-biased genes in the X. Neo-X chromosome analysis also showed that other mechanisms such as gene gain and loss contributed to the paucity of X-linked male-biased genes [20]. The loss mechanism, for instance, could be obtained by intermediate pseudogenization of X-linked copies after gene duplication. In contrast, switch of expression profile between sexes (a male-biased gene turn to a female-biased gene) seems to be uncommon during the *Drosophila* gene evolution [20].

The impact of MSCI on the chromosomal distribution of male-biased genes in *Drosophila* can also be compared to the effect of MSCI in other organisms. In mammals, meiotic X inactivation affects the chromosomal distribution of testis-biased genes [37],[38]. A global gene expression analysis in different spermatogenic phases demonstrated that genes expressed during mitotic phases are enriched on the X chromosome, whereas those expressed later in spermatogenesis are enriched on autosomes [38]. Another study [37] demonstrated, through expression analyses along different spermatogenic stages that autosomal retrogenes specifically compensate for their X-linked parental gene that is silenced during meiosis.

Furthermore, although *Drosophila* and mammalian sex-chromosomes originated independently [39], there are similarities and differences among the forces shaping male gene evolution in the two systems [40]. In the case of sexual antagonism, the chromosomal distribution of male-biased genes in mammals could reflect the role of recessive alleles, since male genes expressed in mitosis tend to accumulate in the X chromosome [38]. In *Drosophila*, however, antagonistic forces acting on dominant mutations may contribute to demasculinization of the X chromosome, as revealed by somatic male-biased gene analyses [3],[20]. Note, however, that there many significant differences between flies and mammals (e.g., mechanisms of dosage compensation) [40]. As such, the statement that dominant/recessive sexual antagonistic alleles govern the chromosomal distribution in genes expressed in somatic cells could be an oversimplification. For genes expressed in meiotic cells, however, MSCI seems to play a similar role in both mammals and flies [37],[38 and this work] by driving the evolution of retrogenes and testis-biased genes. In summary, retrogene expression compensates parental X-linked inactivation and only late spermatogenic genes are depleted on the X. Therefore, taking mammalian and *Drosophila* data together, MSCI may be considered as a general mechanism and force impacting the evolution of testis-expressed genes.

## Material and Methods

### Isolation of tissue samples

All experiments used a wild-type strain of *D. melanogaster* originally collected near Tempe, Arizona (Wolbachia free strain [41]). Cells enriched for mitotic, meiotic and post-meiotic phases were obtained by dissection of apical, proximal and distal regions of the testis, respectively (Figure 1). Testes without seminal vesicles were dissected in PBS. Paired testes were separated using 0.25 mm diameter insect pins and only a single region was dissected from individual testis (Figure 1). This greatly increased the number of necessary dissections, but helped minimize contamination. Apical cells were obtained by separation of apical tips, whereas distal cells were obtained from the detachment of basal regions in order to avoid contamination with spermatocytes (Figure 1). Proximal cells were obtained as follows. First, individual testes placed in small drops had their distal region removed to allow the release of spermatid bundles, reducing the internal turgor in the testis. Then, the apical regions were removed and the spermatocytes were obtained by applying gradual pressure to the middle regions (proximal to the apical tip) in a posterior-anterior direction. During the procedure, contamination from distal regions was avoided by perpendicular positioning of the insect pin to prevent the exit of any remaining spermatid bundles as spermatocytes were teased out of the testis shealth. Cells from the different dissected regions were carefully pipetted to microcentrifuge tubes. For each of three replicate experiments, 250–500 testis dissections were used for RNA isolation. (For more detailed graphic protocols of dissecting testis expression data, see the database we prepared entitled “SpermPress: *Drosophila* spermatogenesis database” on the website (http:// pondside.uchicago. edu/∼longlab/spermpress).

### Immunostaining techniques

Apical, proximal and distal regions of the testis were fixed in 95% ethanol followed by formaldehyde as previously described [42]. Indirect immunofluorescence staining was carried out using mouse α -tubulin (Sigma) and Cy3 donkey anti-mouse IgG (Jackson Laboratories) as primary and secondary antibodies, respectively. DNA was stained with Sytox Green (Amersham). Cells were incubated for 1 h at room temperature in solution containing 1%BSA, 1∶1500 and 1∶500 dilutions for primary and secondary antibodies, respectively. 2 mg/ml RNAse A was included in the primary antibody solution. Fixation and antibody stains were always followed by three wash steps in PBS-T. DNA stained in Sytox green (10 µM solution for 10 min at room temperature) was briefly washed before mounting on slides for visualization with a confocal microscope using Pro-long anti-fade media (Molecular Probes).

### Arrays

Total RNA was extracted from the apical, proximal and distal germ cell populations using PicoPure™ RNA Isolation Kit (Arcturus). Three biological replicates were hybridized into Affymetrix Gene Chip *Drosophila* Genome 2.0 Arrays. cDNAs were synthesized according to the Invitrogen SuperScript RNA Amplification System, except for a modified *in vitro* transcription step where Biotin-Labeled cRNA was produced. Hybridization, scan and data processing were done using Affymetrix default protocols. Gene product expression was measured by hybridization intensity (log2) obtained using RMA background correction and quantile normalization (Bioconductor package in R). Full platform descriptions and data are available at the GEO under accession GSE18502. Individual gene expression profile can also be obtained at SpermPress database (http://pondside.uchicago.edu/~longlab/spermpress).

### Selection of genes expressed in testis (FlyAtlas)

Confirmation of MSCI was done using only genes already known to be expressed in testis (Figure 3). We selected approximately nine thousand gene products present in at least 3 out of 4 *Drosophila* testis microarrays (Presence Call in Flyatlas [43], Table S1).

### Testis-biased genes selection

Testis-biased genes were selected from a testis vs. ovary comparison contained in the Sebida database [44]. We selected 2608 genes that were classified as testis-biased genes in *Drosophila* gonad comparison datasets [3],[4] (Table S3). Testis-biased genes were assigned to 2268 and 331 gene product probe identifications located in the autosomes and in the X chromosome, respectively.

### Parental-retrogene selection

Parental-retrogene pairs were selected from Bai and co-authors [10]. We were able to assign 91 pairs of probe IDs that corresponded to parental-retrogene pairs, excluding 3 pairs of duplication events after retrotransposition [10] (Table S2). We classified parental-retrogene pairs into retroposed “X→A” (n = 27), “A→A” (n = 52), “X→X” (n = 2) and “A→X” (n = 10) groups.

### Statistical analyses

MSCI confirmation. In order to confirm X inactivation, we analyzed expression separately for X-linked and autosomal-linked genes. First, we compared meiotic gene expression to mitotic and to post-meiotic expressions, respectively. We could therefore classify genes as having higher (over), lower (under) or equal expression levels in meiosis compared to the other spermatogenic phases (mitosis or post-meiosis). Simultaneously, the proportions of genes in each class (over-, under- and equal expression) were estimated for X- and autosomal-linked genes. MSCI was detected by observing an excessive number of X-linked genes under-expressed in meiosis relative to any other phase. Excessive number in this case means a significantly higher proportion compared to autosomal-linked genes. In other words, we hypothesized that the X chromosome would possess more genes under-expressed in meiosis as compared to autosomes. Bayesian models were developed to estimate both chromosomal distributions as well as proportions of genes in expression classes [32],[45],[46]. More details about Bayesian estimation (Figure S1) and classification can be found in Model A in Statistical Analyses, Methods in Text S1.

### Retrogene analysis

In order to verify if MSCI affects retrogene movement, we compared the spermatogenic expression of X->A and A->A parental-retrogene pairs. We expected that X->A pairs would have a higher proportion of complementary expression than A->A pairs. Complementary expression was defined as the under-expression of parental gene and the over-expression of the retrogene in meiosis relative to mitosis. Complementary expression was assessed by comparing mean expression intensities between mitosis and meiosis, which were jointly estimated [30],[45]. More specifically, we estimated the probability that a given pair showed meiotic retrogene over-expression and meiotic parental gene under-expression. All gene pairs in each group (X→A and A→A) were used simultaneously in our model estimation, therefore avoiding the need for multiple hypothesis testing corrections. More details about the Bayesian estimation of complementary expression [30],[45] can be found in Model B in Statistical Analyses, Methods in Text S1.

### Testis-biased gene analyses

Two analyses were performed using the testis-biased gene dataset. First, we investigated MSCI by analyzing the proportions of testis-biased X-linked and autosome-linked genes under-expressed in meiosis (Figure 4). We used the under-expressed classes defined by Bayesian model A (Statistical Analyses, Methods in Text S1). Second, we assessed the testis-biased chromosomal proportion for mitotic and meiotic phases (Figure 7). Genes having higher expression in mitosis than in meiosis were considered as mitotic genes (Figure 7B), whereas those having higher expression in meiosis were considered as meiotic genes (Figure 7C). All testis-biased genes chromosomal proportions were parsed from the Sebida database [3],[4],[44]. Testis-biased gene chromosomal proportions were measured relative to the total number of genes by chromosome, where autosomes were pooled together.

## Supporting Information

Figure S1 Bayesian estimation model for differential expression distributions. (A) Differential expression between meiosis and mitosis model through a mixture of two normal distributions (red and black lines). The first normal distribution (red) has a small variance, whereas the second (black) has a significant larger variance. (B) Regions for differential expression classes (over, under and equally) along the mixture distribution.
(0.55 MB TIF)

Figure S2 Pairwise plot for spermatogenic phase expression. Pairwise plots of gene product intensities (lower panel) and correlations (upper panel). Mit, Mei, and Pos correspond to the spermatogenic phases, with three replicates within each phase.
(0.91 MB TIF)

Figure S3 Spermatogenic gene expression analysis for Bayesian Model A and for twofold change method. Scatter plots of intensities (log2) of X-linked (A) and autosomal-linked genes (B) in meiosis versus mitosis comparison. The twofold and Bayesian cutoffs are indicated by blue and pink lines, respectively. (C) and (D) Proportions of genes classified as over-, under-, and equally expressed in meiosis (Bayesian 95% Confidence Intervals also shown). Classifications were made using Bayesian Model A (C) and the twofold intensity change method (D). Note that the twofold change method classifies ∼70% of the data as equally expressed and therefore does not detect differences between X chromosome and autosomal gene proportions. On the other hand, the Bayesian Model is able to detect that a significant proportion of X-linked genes has reduced expression in meiosis, but not in mitosis (Bayesian P≤0.001). Meiosis versus post-meiosis comparison produces similar results.
(0.75 MB TIF)

Figure S4 Distribution of fold expression differences. Boxplot of fold expression (mitotic/meiotic) for genes under expressed in meiosis. Note that the range of expression-fold differences is large.
(0.50 MB TIF)

Figure S5 Spermatogenic gene expression for X-linked and autosomal-linked genes in meiosis versus post-meiosis comparisons. Proportions of genes and their respective Bayesian 95% Confidence Intervals in each of the following classes: (A) Genes over-expressed in meiosis (expression in meiosis greater than expression in post-meiosis); (B) Genes under-expressed in meiosis (expression in meiosis less than expression in post-meiosis). For all comparisons (A) and (B), X chromosome gene proportions are significantly different than autosomal gene proportions (*P stands for Bayesian P, Methods in Text S1).
(0.64 MB TIF)

Table S1 Expression intensities (log2) for all 18801 *D. melanogaster* gene products and their respective classification as over-, under-, or equally expressed in meiosis.
(5.51 MB XLS)

Table S2 Gene product intensities during mitosis and meiosis for 91 parental-retrogene pairs and their respective posterior probability of having complementary expression.
(0.12 MB XLS)

Table S3 Gene product intensities during mitosis and meiosis for 2,599 testis biased gene products and their respective classification as over-, under-, or equally expressed in meiosis.
(0.63 MB XLS)

Text S1 Supplementary methods, list of supplementary tables, references for supplementary methods.
(0.10 MB DOC)
