# Supplementary figures and images for: Stage-Specific Expression Profiling of Drosophila Spermatogenesis Suggests that Meiotic Sex Chromosome Inactivation Drives Genomic Relocation of Testis-Expressed Genes
Source: PLoS Genet. 2009 Nov 20;5(11):e1000731. doi: 10.1371/journal.pgen.1000731 (PMC2770318; doi:10.1371/journal.pgen.1000731)

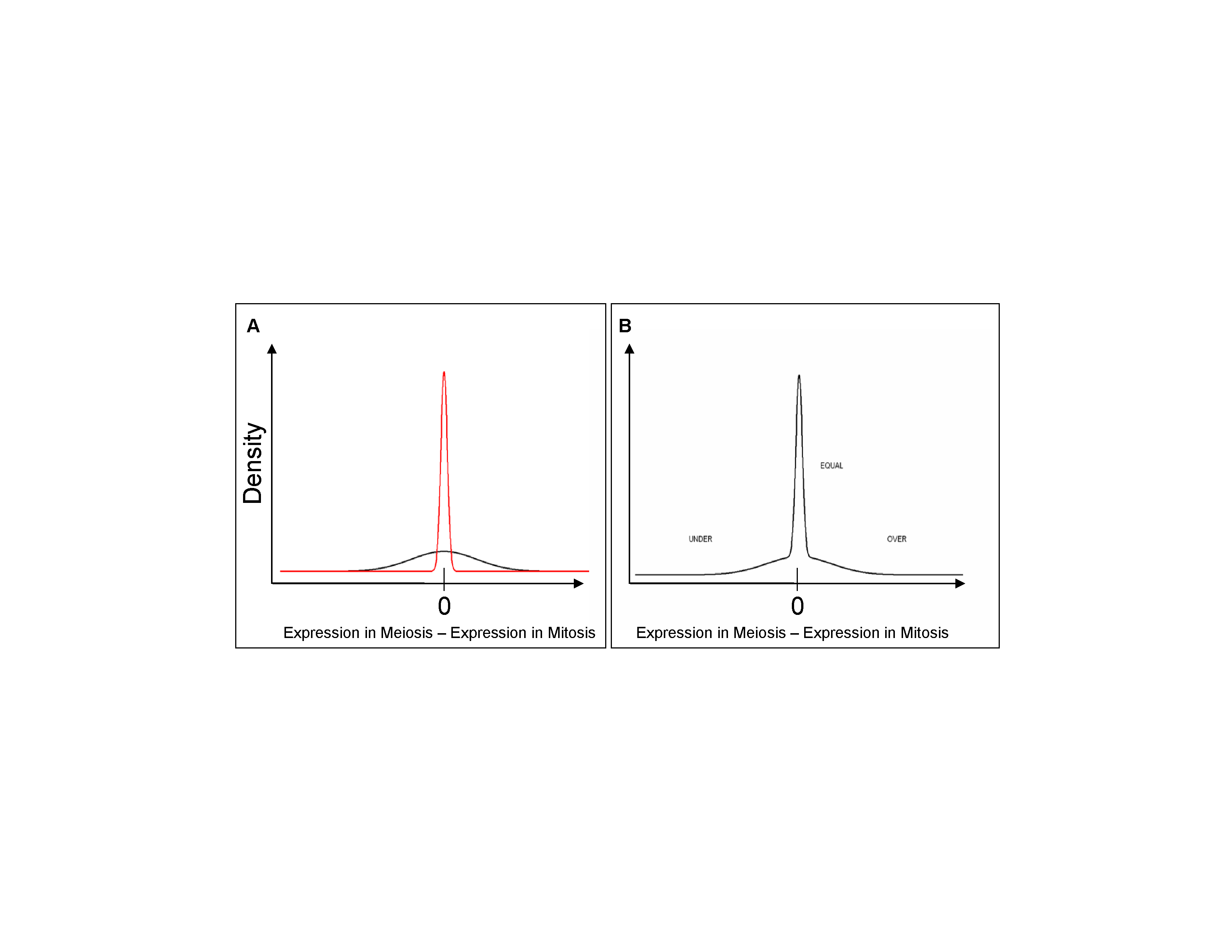

Supplement: Figure S1 — Bayesian estimation model for differential expression distributions. (A) Differential expression between meiosis and mitosis model through a mixture of two normal distributions (red and black lines). The first normal distribution (red) has a small variance, whereas the second (black) has a significant larger variance. (B) Regions for differential expression classes (over, under and equally) along the mixture distribution. (0.55 MB TIF) [file pgen.1000731.s001.tif]

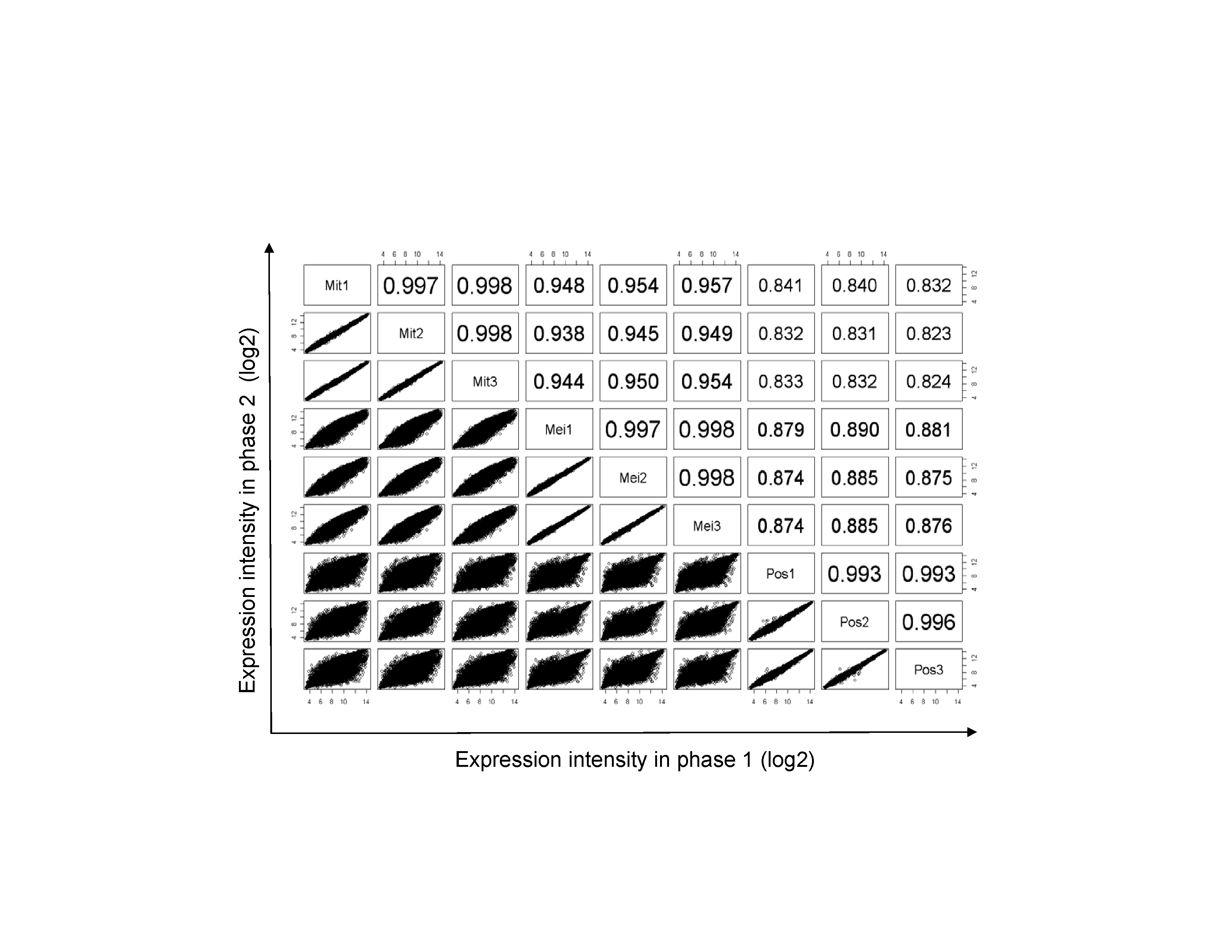

Supplement: Figure S2 — Pairwise plot for spermatogenic phase expression. Pairwise plots of gene product intensities (lower panel) and correlations (upper panel). Mit, Mei, and Pos correspond to the spermatogenic phases, with three replicates within each phase. (0.91 MB TIF) [file pgen.1000731.s002.tif]

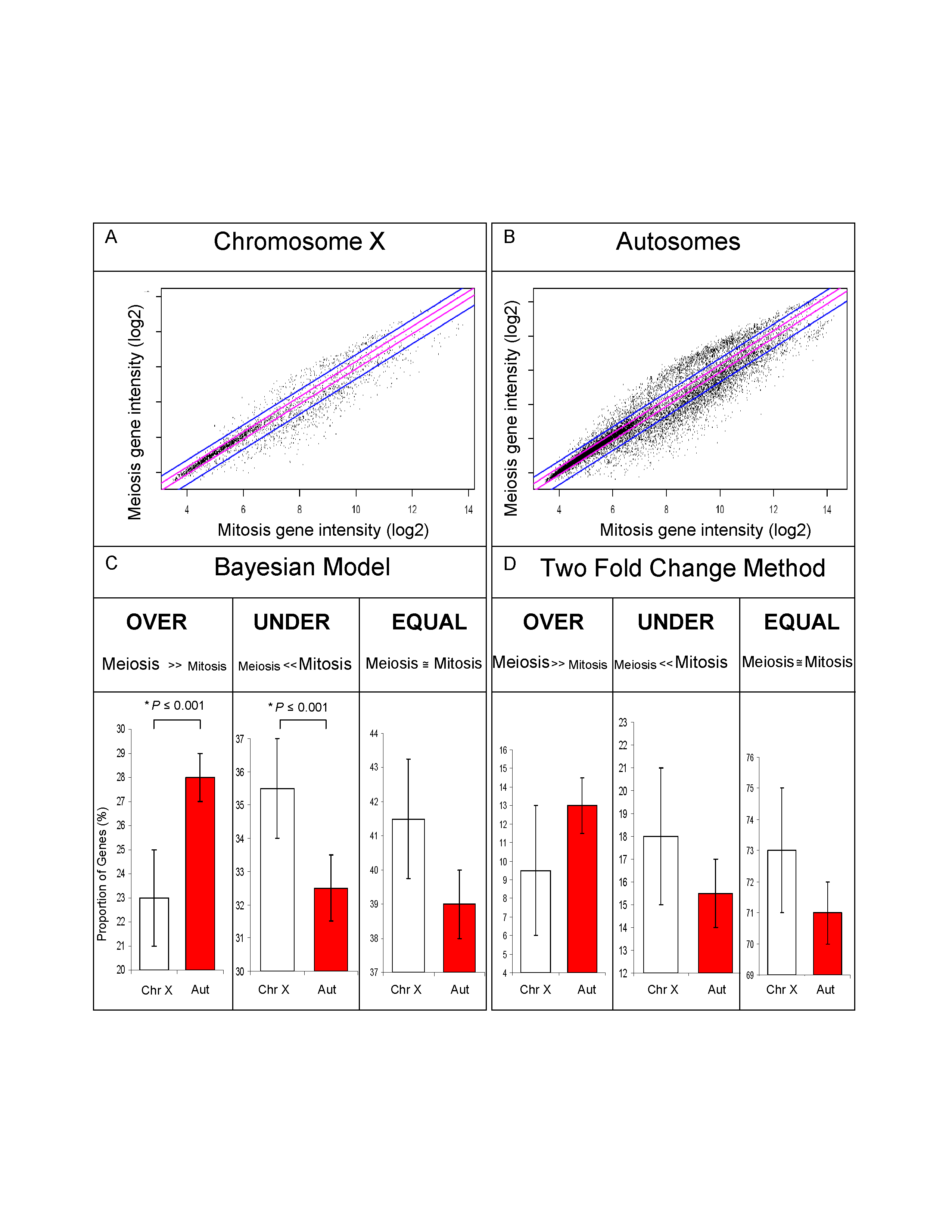

Supplement: Figure S3 — Spermatogenic gene expression analysis for Bayesian Model A and for twofold change method. Scatter plots of intensities (log2) of X-linked (A) and autosomal-linked genes (B) in meiosis versus mitosis comparison. The twofold and Bayesian cutoffs are indicated by blue and pink lines, respectively. (C) and (D) Proportions of genes classified as over-, under-, and equally expressed in meiosis (Bayesian 95% Confidence Intervals also shown). Classifications were made using Bayesian Model A (C) and the twofold intensity change method (D). Note that the twofold change method classifies ∼70% of the data as equally expressed and therefore does not detect differences between X chromosome and autosomal gene proportions. On the other hand, the Bayesian Model is able to detect that a significant proportion of X-linked genes has reduced expression in meiosis, but not in mitosis (Bayesian P≤0.001). Meiosis versus post-meiosis comparison produces similar results. (0.75 MB TIF) [file pgen.1000731.s003.tif]

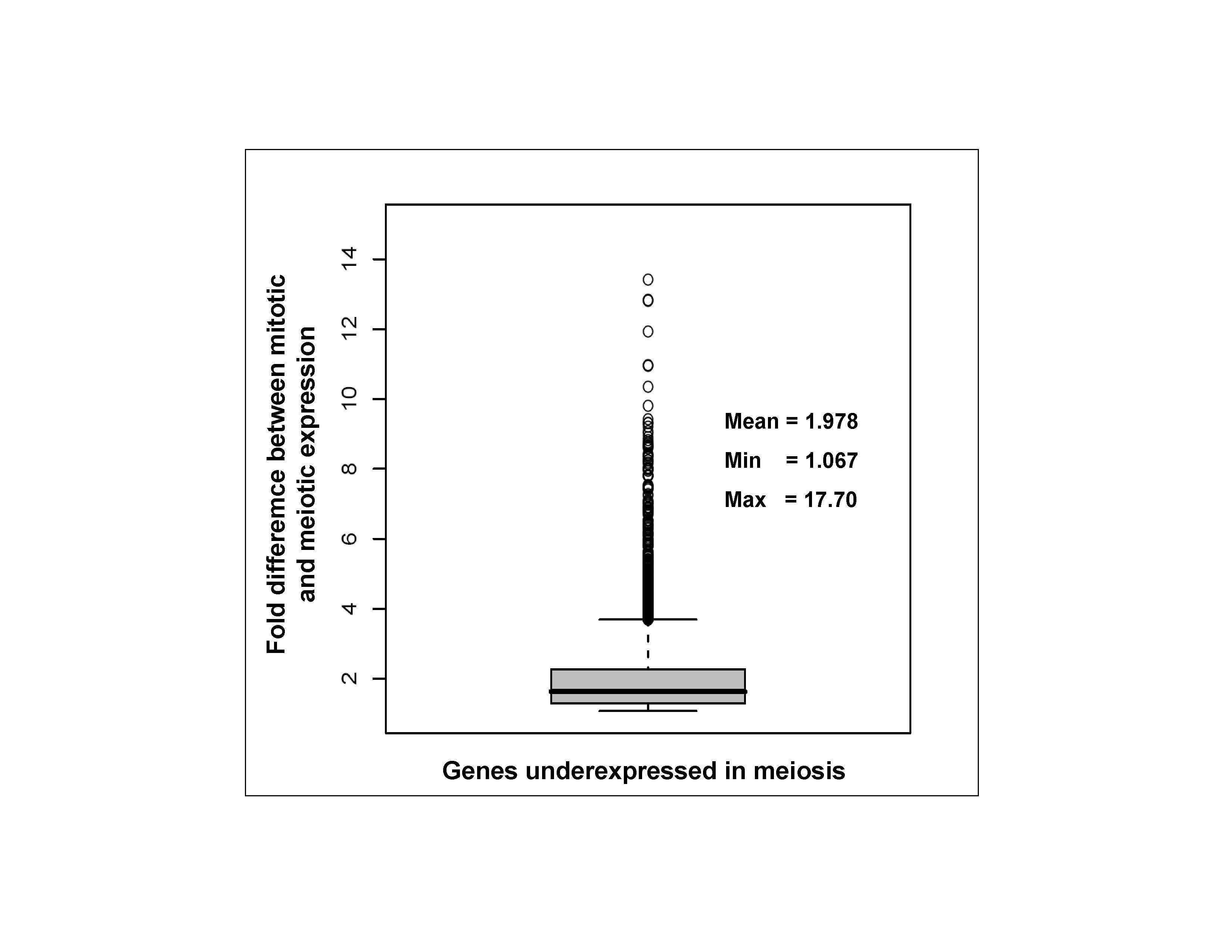

Supplement: Figure S4 — Distribution of fold expression differences. Boxplot of fold expression (mitotic/meiotic) for genes under expressed in meiosis. Note that the range of expression-fold differences is large. (0.50 MB TIF) [file pgen.1000731.s004.tif]

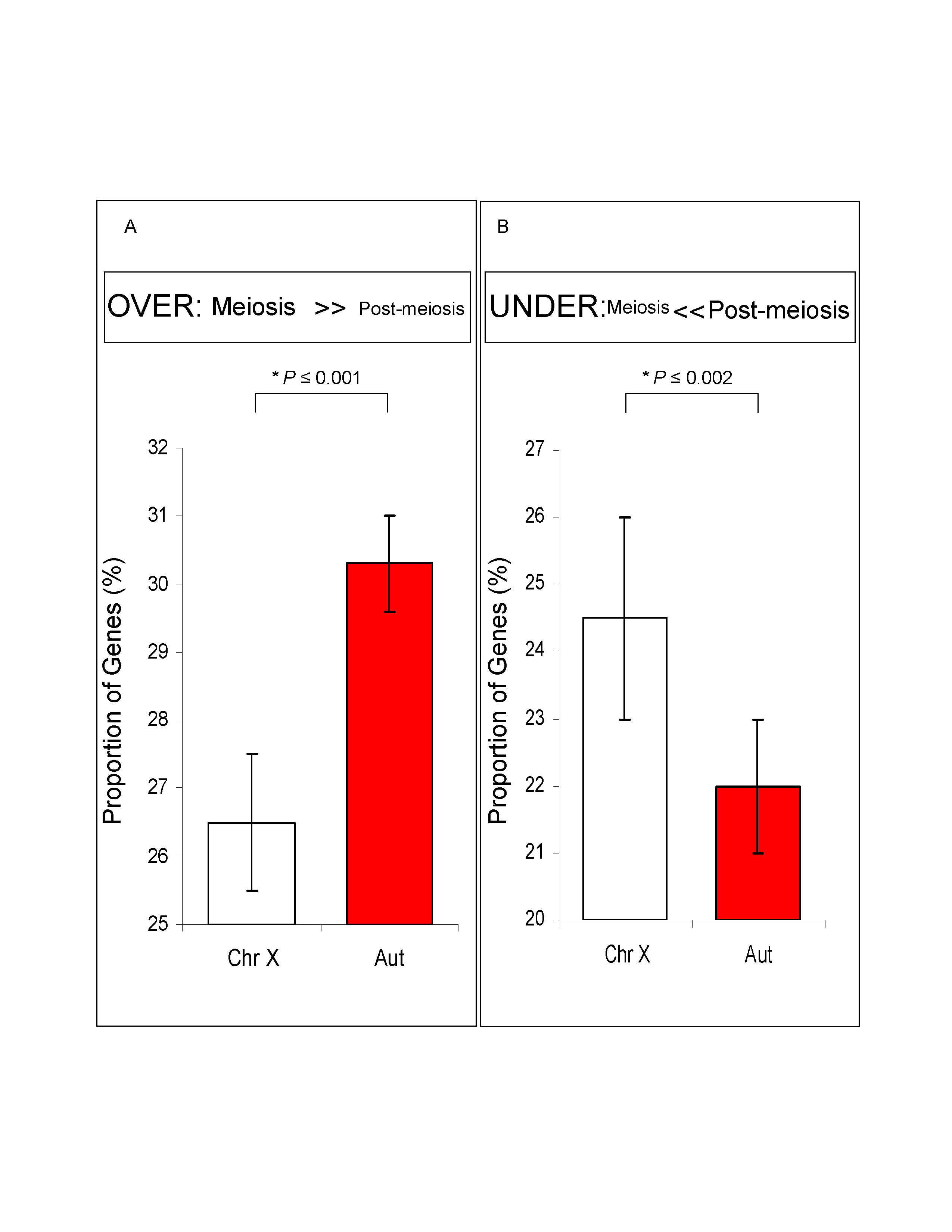

Supplement: Figure S5 — Spermatogenic gene expression for X-linked and autosomal-linked genes in meiosis versus post-meiosis comparisons. Proportions of genes and their respective Bayesian 95% Confidence Intervals in each of the following classes: (A) Genes over-expressed in meiosis (expression in meiosis greater than expression in post-meiosis); (B) Genes under-expressed in meiosis (expression in meiosis less than expression in post-meiosis). For all comparisons (A) and (B), X chromosome gene proportions are significantly different than autosomal gene proportions (*P stands for Bayesian P, Methods in Text S1). (0.64 MB TIF) [file pgen.1000731.s005.tif]
