# Supplementary material for: Stage-Specific Expression Profiling of Drosophila Spermatogenesis Suggests that Meiotic Sex Chromosome Inactivation Drives Genomic Relocation of Testis-Expressed Genes
Source: PLoS Genet. 2009 Nov 20;5(11):e1000731. doi: 10.1371/journal.pgen.1000731 (PMC2770318; doi:10.1371/journal.pgen.1000731)
Supplement: Text S1 — Supplementary methods, list of supplementary tables, references for supplementary methods. (0.10 MB DOC) [file pgen.1000731.s009.doc]

Stage-specific expression profiling of *Drosophila* spermatogenesis suggests that meiotic sex chromosome inactivation drives genomic relocation of testis-expressed genes

Maria D. Vibranovski, Hedibert F. Lopes, Timothy L. Karr, Manyuan Long^*^

*To whom correspondence should be addressed: mlong@midway.uchicago.edu

Supplementary Materials and Methods document includes:

1. Supplementary Methods
2. List of Supplementary Tables
3. References for Supplementary Materials and Methods

**1. Supplementary Methods**

**1.1 Statistical Analyses**

Our statistical analyses are composed of two Bayesian models (A and B). Model A was developed to assess MSCI and then used to evaluate the proportion of testis-biased genes expressed in mitosis and meiosis. Model B was specifically developed to estimate and compare proportions of complementary expression in groups of parental-retrogene pairs.

**1.1.1. Model A**: mixture of normal densities model to assess differences in gene expression between spermatogenic phases.

Meiotic sex chromosome inactivation was assessed by comparing gene product expression between meiosis and the other two spermatogenic phases. Differences in hybridization intensities between spermatogenic phases were estimates for X chromosome and autosomes, separately. Gene differences were based on average intensities over three replicates since within gene variability is negligible when compared to between gene variability [1, 2]. Genes were then classified as over-, under- or equally expressed in meiosis relative to mitosis or post-meiosis. Simultaneously, the proportions of genes in each class were estimated for X- and autosomal-linked genes. X inactivation was detected as an excessive number of X-linked genes under-expressed in meiosis relative to any other phase. Excessive number means a significantly higher proportion compared to autosomal-linked genes.

In order to classify genes as over-, under- or equally expressed in meiosis, we estimated the X chromosome and autosomal differential expression distributions for meiosis and both other spermatogenic phases, e.g., distribution of gene expression (in meiosis) minus (in mitosis) (Figure S1). The differential expression distributions were estimated through Bayesian analysis performed using a two-component mixture of normal distributions model (Figure S1A). Specifically, posterior estimation of the mixture components means, variances and weights is performed via Markov Chain Monte Carlo algorithms [3].

The two distribution means are expected to be close to zero, whereas the variance of the first normal distribution is supposed to be significant smaller than the variance of the second normal distribution (Figure S1A). Genes with differential expression within the first normal distribution were considered as equally expressed, whereas genes within the second normal distribution were classified as over- or under-expressed depending on the positive or negative value of their expression differences (Figure S1B).

In more detail, for a given chromosome type (X or autosome), let $x_{gpr}$be the r^th^ intensity replication in phase p (mitosis, meiosis or post-meiosis) of gene product g and $\bar{x}_{gp}$be the average intensity over the three replicates. For mitosis (phase 1) and meiosis (phase 2), for instance, the difference in expression intensities, defined by$d_{g}=\bar{x}_{g2}-\bar{x}_{g1}$, is modeled as

$$d_{g}\sim\pi N(\delta_{1},\tau_{1}^{2})+(1-\pi)N(\delta_{2},\tau_{2}^{2})$$

with mean differences $\delta_{1}$ and $\delta_{2}$expected to be close to zero, while $\tau_{1}^{2}$is expected to be significantly smaller than $\tau_{2}^{2}$ to reflect an excess of negligible differential expression in the first component of the mixture (Figure S1A). The weights $\pi$ and 1-$\pi$ represent the proportions of genes equally and non-equally expressed, respectively. Within the group of non-equally expressed genes, a gene g is classified as over-expressed if, in addition, $d_{g}>0$, or as under-expressed if $d_{g}<0$ (Figure S1B and Figure 3A and 3B for meiosis - mitosis and Figure S5A and S5B for meiosis - post-meiosis). The predictive distribution of differential expression for a new gene is obtained by averaging the above mixture with respect to the joint posterior distribution of the model parameters [3]. Marginal posterior distributions of model parameters, based on relatively uninformative prior distributions, are available upon request.

Instead of deterministically classifying genes as equal, over- or under-expressed in meiosis, our modeling strategy assigns to each gene the probability of being placed into a particular class (column “Mei:Mit_BPP” in Table S1). In most cases, these probabilities are either 0 or 1, which would lead to the simple categorization of genes into the three classes. However, for most genes (60% of genes) these probabilities are spread across classes. Therefore, the final proportions of genes within each class are themselves uncertain, as the Bayesian 95% confidence intervals indicate (Figure 3 and 4; Figures S3 and S5).

**1.1.2. Model B:** Bayesian hierarchical model for complementary expression in retrogenes

Model B was specifically developed to estimate and compare proportions of complementary expression in groups of parental-retrogene pairs. Complementary expression is defined as under expression of the parental gene and over-expression of the retrogene, in meiosis relative to mitosis. Parental-retrogenes complementary expression is a function of mean intensities, which in turn are jointly estimated by a Bayesian hierarchical model (B) [2,4].

Two chromosome groups were defined, one with genes retroposed from the X chromosome and another with genes retroposed from autosomes. For each gene product g in the same group, let $x_{gspr}$be the r^th^ intensity replication in phase p (p=1:mitosis or p=2:meiosis) and gene type s (s=1:parental copy or s=2:retrogene).

The model for $x_{gspr}$is

$$\begin{matrix} \left(\begin{matrix} x_{gs1r} \\ x_{gs2r} \end{matrix}\right)\sim N\left[\left(\begin{matrix} \theta_{gs1} \\ \theta_{gs2} \end{matrix}\right),\left(\begin{matrix} \sigma^{2} & 0 \\ 0 & \sigma^{2} \end{matrix}\right)\right] \\ \left(\begin{matrix} \theta_{gs1} \\ \theta_{gs2} \end{matrix}\right)\sim N\left[\left(\begin{matrix} \theta_{s1} \\ \theta_{s2} \end{matrix}\right),\left(\begin{matrix} \tau_{1}^{2} & 0 \\ 0 & \tau_{2}^{2} \end{matrix}\right)\right] \end{matrix}$$

The first level models the gene product intensities between the two phases, while the second level models the gene product intensity means across genes in the X chromosome or autosomes. Marginal posterior distributions of model parameters, based on relatively uninformative prior distributions, are available upon request. Therefore, the probability of complementary expression in gene g is computed from our model as$\Pr(\theta_{g11}>\theta_{g12},\theta_{g21}<\theta_{g22})$ (Figures 6A and 6B). We examine the proportion of genes with complementary expression (the above probability being larger than 50%) in both chromosome groups (Figure 5).

Once again, instead of deterministically classifying genes as having complementary expression or not, our modeling strategy assigns to each gene the probability of being placed in either of the two classes (column “Comp_BPP” in Table S2). It is very interesting that most of the X→A parental-retrogene pairs (Figure 6A) show a probability of either zero or one for complementary expression. Those probabilities reflect the lower uncertainty attached to classification of a gene pair as having complementary expression or not. On the other hand, several A→A parental-retrogene pairs (Figure 6B) have probabilities that are markedly different from zero or one (e.g., 0.7 and 0.4). These uncertainties when classifying genes directly affects the confidence intervals, which are much larger for A→A than for X→A parental-retrogene pairs (Figure 5C).

**1.1.3 Bayesian P**

In this work, Bayesian P stands for the probability that two chromosomal proportions are equal. More specifically, Bayesian P stands for the probability of a particular hypothesis, in general represented by P((Z,W) in H), where Z and W are the measures under study and H the hypothesis. For instance, when Z and W are, respectively, the proportion of over-expressed X- and autosome-linked genes in meiosis, and the hypothesis is that Z and W are the same, and the Bayesian P is Pr(Z>W).

**2. Supplementary Figures**

**2.1 List and description of Supplementary Figures**

- Figure S1 presents the Bayesian estimation model for differential expression distributions.
- Figure S2 provides pairwise plots of spermatogenic expression, including correlations between spermatogenesis phases.
- Figure S3 compares spermatogenic gene expression analyses for X-linked and autosomal genes using Bayesian Model A (Supplementary Methods) and the twofold change method.
- Figure S4 provides the Boxplot of fold expression (mitotic/meiotic) for genes under-expressed in meiosis.
- Figure S5 Spermatogenic gene expression for X-linked and autosomal-linked genes in meiosis vs. post-meiosis comparison.

**3. Supplementary Tables**

Tables S1 to S3 are available as separate files.

**3.1 List and description of Supplementary Tables**

- Table S1 lists expression intensities (log2) for all 18801 *D*. *melanogaster* gene products and their respective classification as over-, under- or equally expressed in meiosis.
- Table S2 presents gene product intensities during mitosis and meiosis for 91 parental-retrogene pairs and their respective posterior probability of having complementary expression.
- Table S3 presents gene product intensities during mitosis and meiosis for 2599 testis-biased gene products and their respective classification as over-, under- or equally expressed in meiosis.

**3.2 General description of columns in the Tables S1 to S3** (All affymetrix information was obtained from Drosophila_2.na21.annot.cvs file)

- **Probe_Set_ID:** Affymetrix identification for probe set.
- **Representative_Public_ID:** public identification of a gene product according to Affymetrix.
- **Gene_Symbol:** gene symbol according to Affymetrix (Tables S1 and S3).
- **Alignments_Chr:** Probe Chromosomal localization (Affymetrix).
- **Alignments_pos:** position mapped on to the chromosome according to Affymetrix.
- **Mit1, Mit2, Mit3, Mit_mean:** gene product intensity (log2) in mitosis (three replicates and mean).
- **Mei1, Mei2, Mei3, Mei_mean:** gene product intensity (log2) in meiosis (three replicates and mean).
- **Pos1, Pos2, Pos3:** gene product intensity (log2) in post-meiosis (three replicates).
- **Mei:Mit_BC**: Bayesian classification of expression in meiosis in relation to mitosis: Over, Under or Equal (*e.g.*, *Over* means that a gene has higher expression in meiosis than in mitosis).
- **Mei:Mit_BPP:** Bayesian posterior probability of being equally expressed in meiosis in relation to mitosis. A posterior probability lower than 0.5 means that the gene is not equally expressed.
- **Mei:Pos_BC:** Bayesian classification of expression in meiosis in relation to post-meiosis (*e.g.*, *Over* means that a gene has higher expression in meiosis than in post-meiosis).
- **Mei:Pos_BPP:** Bayesian posterior probability of being equally expressed in meiosis in relation to post-meiosis. A posterior probability lower than 0.5 means that the gene is not equally expressed.
- **FlyAtlas:** YES or NO for genes with presence call in Flyatlas testis microarrays.
- **Pair_no:** Parental-retrogene pair number.
- **Pair_name:** Parental-retrogene pair name.
- **Movement:** Retroposition direction (e.g., X→ stands for retroposition from the X chromosome).
- **Parental_Pb:** Parental probe set identification.
- **Retrogene_Pb:** Retrogene probe set identification.
- **Comp_BPP:** Bayesian posterior probability of complementary expression. A posterior probability greater than 0.5 means that the gene has complementary expression.

**4. References**

1. Müller P, Parmigiani G, Rice K (2006) FDR and Bayesian multiple comparisons rules. In: Bernardo JM, Bayarri MJ, Berger JO, Dawid AP, Heckerman D, Smith AFM, West M, editors. Bayesian Statistics 8 (with discussion). Oxford: Oxford University Press. pp. 349-370.
2. Do K-A, Müller P, Vannucci M (2006) Bayesian Inference for Gene Expression and Proteomics. Cambridge: Cambridge University Press.
3. Gamerman D, Lopes HF (2006) Markov Chain Monte Carlo: Stochastic Simulation for Bayesian Inference (2nd Edition). Boca Raton: Chapman & Hall/CRC.
4. Lopes HF, Müller P, Ravishanker N (2007) Bayesian computational methods in biomedical research. In: Khattree R, Naik DN, editors. Computational Methods in Biomedical Research. Boca Raton: Chapman & Hall/CRC. pp. 211-259.
